# Antisepsis and Antiseptics

**Published:** 1895-09

**Authors:** 


### BOOK NOTICES.

**Antisepsis and Antiseptics.** By Charles Milton Buchanan, M. D., Professor of Chemistry, Toxicology, and Metallurgy, National University, Washington, D. C., with an introduction by Professor Augustus C. Bernays. The Terhune Company, publishers of medical books. Newark, N. J. 1895. Price, $1.25.

This remarkable little volume is a perfect encyclopedia of the whole subject of antisepsis. It contains the history of the antiseptic idea from the earliest times down to the present. It then proceeds to give the philosophy and scientific explanation of the whole germ theory and the reason for the practice of antisepsis. Then comes a description of the relation of antiseptics to general medicine, surgery, obstetrics, and gynecology. A list of all the antiseptics known, with statement of composition of each one, and estimate of its value as a germicide is given in detail, all arranged with the utmost care and attention to completeness of description. No matter whether the reader of this notice is a believer in the germ theory or not, he should have this little book; for every accomplished doctor wishes to know what is taught. In this book he is certain to get the latest information concisely stated, and that, too, with the practical details all fully set forth. As before stated, it is a veritable encyclopedia, with the statements well condensed, so that the book is only a duodecimo of 350 pages.

Different books noticed in these pages have been recommended for their treatment of the germ theory; but this treatment was a scientific exposition of its principles. The present book treats it from the practical side, giving the character of the substances used, their active value, and thus affording a choice in the selection of any antiseptic for any specified purpose.

